# The Forty-Sixth Euro Congress on Drug Synthesis and Analysis: Snapshot †

**DOI:** 10.3390/molecules22111848

**Published:** 2017-10-28

**Authors:** Pavel Mucaji, Atanas G. Atanasov, Andrzej Bak, Violetta Kozik, Karolina Sieron, Mark Olsen, Weidong Pan, Yazhou Liu, Shengchao Hu, Junjie Lan, Norbert Haider, Robert Musiol, Jan Vanco, Marc Diederich, Seungwon Ji, Jan Zitko, Dongdong Wang, Danica Agbaba, Katarina Nikolic, Slavica Oljacic, Jelica Vucicevic, Daniela Jezova, Anna Tsantili-Kakoulidou, Fotios Tsopelas, Constantinos Giaginis, Teresa Kowalska, Mieczyslaw Sajewicz, Jerzy Silberring, Przemyslaw Mielczarek, Marek Smoluch, Izabela Jendrzejewska, Jaroslaw Polanski, Josef Jampilek

**Affiliations:** 1 Department of Pharmacognosy and Botany, Faculty of Pharmacy, Comenius University, Odbojarov 10, 83232 Bratislava, Slovakia; 2 Institute of Genetics and Animal Breeding of the Polish Academy of Sciences, Postepu 36A, 05-552 Jastrzebiec, Poland; d.wang@ighz.pl; 3 Department of Pharmacognosy, University of Vienna, Althanstrasse 14, 1090 Vienna, Austria; 4 Institute of Chemistry, University of Silesia, Szkolna 9, 40007 Katowice, Poland; andrzej.bak@us.edu.pl (A.B.); robert.musiol@us.edu.pl (R.M.); teresa.kowalska@us.edu.pl (T.K.); mieczyslaw.sajewicz@us.edu.pl (M.S.); polanski@us.edu.pl (J.P.); 5 Department of Synthesis Chemistry, Faculty of Mathematics, Physics and Chemistry, University of Silesia, Szkolna 9, 40007 Katowice, Poland; violetta.kozik@us.edu.pl; 6 Department of Physical Medicine, Medical University of Silesia, Medykow 18, 40752 Katowice, Poland; ksieron@sum.edu.pl; 7 Department of Pharmaceutical Sciences, College of Pharmacy Glendale, Midwestern University, 19555 N. 59th Avenue, Glendale, AZ 85308, USA; molsen@midwestern.edu; 8 State Key Laboratory of Functions and Applications of Medicinal Plants, Guizhou Medical University, 3491 Baijin Road, Guiyang 550014, China; wdpan@163.com (W.P.); liuyazi19@gmail.com (Y.L.); hushengcao0221@163.com (S.H.); lanjunjie2007@163.com (J.L.); 9 Key Laboratory of Chemistry for Natural Products of Guizhou Province and Chinese Academy of Sciences, 3491 Baijin Road, Guiyang, 550014, China; 10 Department of Pharmaceutical Chemistry, University of Vienna, Althanstraße 14, A-1090 Vienna, Austria; norbert.haider@univie.ac.at; 11 Department of Inorganic Chemistry & Regional Centre of Advanced Technologies and Materials, Faculty of Science, Palacky University, 17. listopadu 12, 77146 Olomouc, Czech Republic; jan.vanco@upol.cz; 12 Department of Pharmacy, College of Pharmacy, Seoul National University, 1 Gwanak-ro, Seoul 08826, Korea; marcdiederich@snu.ac.kr (M.D.); jsw0525@snu.ac.kr (S.J.); 13 Department of Pharmaceutical Chemistry and Pharmaceutical Analysis, Faculty of Pharmacy in Hradec Kralove, Charles University, Heyrovskeho 1203, 50005 Hradec Kralove, Czech Republic; jan.zitko@faf.cuni.cz; 14 Department of Pharmaceutical Chemistry, Faculty of Pharmacy, University of Belgrade, Vojvode Stepe 450, 11221 Belgrade, Serbia; danica@pharmacy.bg.ac.rs (D.A.); knikolic@pharmacy.bg.ac.rs (K.N.); sfilipic@pharmacy.bg.ac.rs (S.O.); jelicav@pharmacy.bg.ac.rs (J.V.); 15 Laboratory of Pharmacological Neuroendocrinology, Institute of Experimental Endocrinology, Biomedical Research Center, Slovak Academy of Sciences, Dubravska cesta 9, 84505 Bratislava, Slovakia; daniela.jezova@savba.sk; 16 Department of Pharmaceutical Chemistry, Faculty of Pharmacy, National and Kapodistrian University of Athens, Panepistimiopolis, Zografou, 15771 Athens, Greece; tsantili@pharm.uoa.gr; 17 Laboratory of Inorganic and Analytical Chemistry, School of Chemical Engineering, National Technical University of Athens, Iroon Polytechniou 9, 15780 Athens, Greece; ftsop@central.ntua.gr; 18 Department of Food Science and Nutrition, School of Environment, University of the Aegean, 81400 Myrina, Lemnos, Greece; cgiaginis@aegean.gr; 19 Department of Biochemistry and Neurobiology, Faculty of Materials Science and Ceramics, AGH University of Science and Technology, Mickiewicza 30, 30059 Krakow, Poland; jerzy.silberring@agh.edu.pl (J.S.); przemyslaw.mielczarek@agh.edu.pl (P.M.); smoluch@agh.edu.pl (M.S.); 20 Department of Crystallography, Faculty of Mathematics, Physics and Chemistry, University of Silesia, Bankowa 12, 40006 Katowice, Poland; izabela.jendrzejewska@us.edu.pl; 21 Department of Pharmaceutical Chemistry, Faculty of Pharmacy, Comenius University, Odbojarov 10, 83232 Bratislava, Slovakia

**Keywords:** natural compounds, drug design and development, medicinal chemistry, organic synthesis, chemical biology, pharmaceutical analysis, new small entities, therapeutic proteins

## Abstract

The 46th EuroCongress on Drug Synthesis and Analysis (ECDSA-2017) was arranged within the celebration of the 65th Anniversary of the Faculty of Pharmacy at Comenius University in Bratislava, Slovakia from 5–8 September 2017 to get together specialists in medicinal chemistry, organic synthesis, pharmaceutical analysis, screening of bioactive compounds, pharmacology and drug formulations; promote the exchange of scientific results, methods and ideas; and encourage cooperation between researchers from all over the world. The topic of the conference, “Drug Synthesis and Analysis,” meant that the symposium welcomed all pharmacists and/or researchers (chemists, analysts, biologists) and students interested in scientific work dealing with investigations of biologically active compounds as potential drugs. The authors of this manuscript were plenary speakers and other participants of the symposium and members of their research teams. The following summary highlights the major points/topics of the meeting.

## 1. Introduction

MucajiP.JampilekJ.

Discoveries within pharmaceutical and biomedical sciences have been increasingly accelerating, therefore communication among scientists and research teams around the world has become more and more important. Beyond participation in various scientific databases and portals and other virtual events, another possibility is attendance at select specialised conferences. The traditional symposium “Drug Synthesis and Analysis” has a long history. Over the last decades it has been transformed from a local Czechoslovak, later Czech and Slovak symposium into a conference with several international keynote speakers to a full-value international conference. Thus, this traditional symposium has changed to a prestigious international conference that can be found in the worldwide calendar of conference events as “EuroCongress on Drug Synthesis and Analysis” (ECDSA) [1]. This original conference and successors are high-quality meetings that cover all the fields of pharmaceutical sciences. Currently, the main aim of ECDSA is to develop a platform for scientific contacts between researchers dealing with pharmaceutical sciences from European and other countries around the world and to support cooperation between scientists in this field. Excellent speakers from all over the world presented their results at individual sessions of the 46th EuroCongress on Drug Synthesis and Analysis (ECDSA-2017) that was held in Bratislava, capital of Slovakia, from 5–8 September 2017 [1].

The start of a two-year programme of study in the academic year 1939–1940 was organised by the Faculty of Arts and the Faculty of Medicine and can be considered an important milestone in the study of modern pharmacy in Slovakia. Beginning in 1948, the length of study was extended to four years in Czechoslovakia. The independent Faculty of Pharmacy at Comenius University was established by a government regulation that came into effect on 1 September 1952 [2]. Thus, during this conference, the Comenius University faculty celebrated the 65th anniversary of its establishment. It was a pleasure and an honour for the faculty to welcome many prestigious scientists as well as many young researchers and students on the faculty grounds.

This year of the ECDSA conference [1] was devoted to the following research topics: (*i*) drug design, research and development; (*ii*) natural compounds; (*iii*) structure, function and interactions of proteins, engineered enzymes; (*iv*) nucleic acids chemistry; (*v*) chiral compounds; (*vi*) structural and solid state analysis; (*vii*) drug formulations and drug delivery systems; (*viii*) chemical biology and biological chemistry; (*ix*) pharmacology and ADMET; (*x*) biomaterials and nanomaterials; (*xi*) drug formulations and drug delivery systems; and (*xii*) pharmaceutical service and history of pharmacy. The aim of the symposium is to promote the exchange of scientific results, methods and ideas and encourage cooperation between researchers from all over the world. In total, 190 active participants from 20 countries around the world presented their novel results.

The authors of this manuscript are plenary speakers and other participants of the symposium and members of their research teams. The following summary highlights major points and topics of the symposium. Individual experimental and review contributions/sections are ordered from in-silico design, synthesis of potential drugs to their analytical characterization and screening of biological activity. The manuscript closes with hot topics focused on counterfeit drug detection and big data problems in drug design.

## 2. Finding In-silico Paradise in Multidimensional QSAR Studies

BakA.KozikV.SieronK.

The rational creation of a desired compound pharmacological profile, not the compound itself, has been a tremendously challenging issue due to the complex nature of the diverse drug–target interactions ruled by inter/intra-molecular phenomena. This challenging object of interest for chemists involved in property-based drug discovery can be illustrated by the balance in the molecule’s pharmacokinetic/pharmacodynamic profile, termed ‘sweet spot,’ between sufficient drug potency and acknowledged constraints on the physicochemical properties of the drug candidate [3]. The art of specifying molecules of potential therapeutic value at the ‘pre-synthesis’ stage can be assisted by computer-aided molecular design (CAMD) techniques for the comprehensive mapping of the compound topology and/or topography into the property-based chemical space.

Modelling multidimensional quantitative structure-activity relationship (mD–QSAR) techniques are pragmatic provided that a congeneric series of compounds is proceeded. The analogy is crucial in chemistry, especially in cases when we do not fully understand the investigated process or effect. In fact, a variety of protocols have evolved from this concept (1D ÷ 7D–QSAR), however their practical importance for drug design is still controversial. The *a priori* calculation of molecular descriptors, crucial for the compound’s bioavailability and hence critical for the prospective drug candidate, is necessary to make predictions of a chosen property profile. The pursued target of in-silico modelling is to produce statistically robust models capable of making accurate quantitative predictions including binding affinity, metabolic fate and pharmacokinetic or toxicity parameters (ADMET) [4]. The molecular ADMET–tailored properties are essentially estimated, based on the molecular structure, as ‘intuitive roadmaps’ even before the synthesis of the molecule has been rationalized. Considerable endeavours have been employed to implement in-silico protocols which resulted in two essential paradigms classified as ‘indirect’ ligand-based (receptor-independent) and ‘direct’ structure-based (receptor-dependent), respectively. Regarding a similar property-principle of the quantitative SAR approach, a ‘reverse’ image of the target binding geometry might be retrieved from the ensemble of intrinsic and/or extrinsic features of structurally-related (bio)active molecules which share the same pharmacological pattern [5,6]. A number of 3D–QSAR procedures have been implemented, however the comparative molecular field analysis (CoMFA) diffused quickly into medicinal and computational chemistry becoming a cornerstone for computer–aided molecular design. A number of alternative CoMFA–like protocols have emerged, e.g., comparative molecular surface analysis (CoMSA) that implemented improvements in the molecular description, alignment rules and, subsequently, the modelling performance (predictive quality) [7].

In the broadest sense, 4D–QSAR methodology has evolved from the molecular shape analysis (MSA) by explicit enhancement of 3D–QSAR approaches, where the substitution of the single–conformer concept with cube–like structures of different resolution produces a fuzzy pattern of molecular objects. The 4D–QSAR formalism successfully incorporates some CoMFA characteristics employing the spatial grid, where the grid cell size is viewed as a ‘methodology parameter’ to produce a molecular shape spectrum (MSS) according to the trial alignment rule [8]. Moreover, the neural version of Hopfinger’s cube formalism, namely SOM–4D–QSAR, employing the Kohonen self–organizing maps has been applied to produce a fuzzy 4D–QSAR–like representation of the conformational space as an appealing alternative which performs comparably to its grid counterpart [9]. Additionally, we concentrated on systematic model space inspection by splitting data collection into training/test subsets to monitor statistical estimator performance in the effort for mapping of the probabilistic pharmacophore geometry using the stochastic model validation (SMV) approach.

The automated variable reduction with the IVE–PLS procedure represents a sieve for detecting only those descriptors that have prescribed the greatest individual weighting for the observed cholic acids analogue activity. The ‘pseudo–consensus’ 4D–QSAR methodology was used to extract an ‘average’ 3D–pharmacophore by exploring various data subpopulations and which embodies the quantity for a quality argument to indicate the relevant contributing factors of the cholic acid absorption activity [10]. A comparative structure-affinity study of anthraquinone dye adsorption on cellulose fibre was performed using receptor-dependent 4D-QSAR methods based on grid and neural (SOM) methodology coupled with IVE-PLS procedure [11]. The applied RD 4D-QSAR approach focuses mainly on the ability of mapping dye properties to verify the concept of tinctophore in dye chemistry. The working paradigm of fragment-based design or fragonomics assumes that well-placed fragments account for crucial ligand-receptor interactions, while the rest of the molecule mainly serves as a scaffold that holds the ‘active’ fragments together. Is it possible to experimentally and/or theoretically tailor protein cavity regions that are important for ligand recognition and specificity, namely a fragment-based domain design? How can even the vague electrostatic, steric or lipophilic patterns that govern changes in binding ligands affinity be specified? Hence, the application of the potential that is represented by libraries of artificial targets binding a given guest molecule in physiological conditions as a model system for the emulation of ligand-receptor interactions has been reported [12].

Lipophilicity is generally regarded as a first–rate physicochemical parameter increasingly relevant in characterizing both the pharmacokinetic (ADMET) and pharmacodynamic aspects of drug–receptor/enzyme interactions, and which often correlates well with the bioactivity of chemicals. Numerous compounds of different chemical structures were evaluated/applied as absorption promoters. Cholic acid is one of the most important human bile acids with a range of pharmacological activities. Various software log *P* predictors for estimation of the numerical lipophilic values for a set of cholic acid derivatives have been employed and subsequently crossed-compared with the experimental parameter (*R*_M_). The mean values of the selected molecular descriptors that average over the chosen calculation methods (consensus clog *P*) were subsequently correlated with the *R*_M_ parameter. To investigate the variations within the ensemble of cholic acid derivatives PCA and SOM procedures were employed to visualize the major differences in the performance of drug promoters with respect to their lipophilic profile [13].

In conclusion, a range of calculation methods were employed to analyse the experimental data using *consensus* methodology for finding in-silico optimization in multidimensional QSAR studies. One should be aware that ‘statistical unicorns exist on paper not in reality’, therefore we should not blindly follow theoretical estimators and sometimes do the experiments [14].

## 3. Computational Prediction of Aspartyl(Asparaginyl)-β-Hydroxylase Substrates in Cancer Signalling Pathways and Implications for Cancer Therapeutics

OlsenM.

Aspartyl(asparaginyl)-β-hydroxylase (ASPH) is a 2-oxoglutarate (2OG) utilizing iron-dependent dioxygenase closely related to epigenetic enzymes such as KDM, TET1-3, and FTO [15]. ASPH catalyzes post-translational hydroxylation of critically positioned aspartic acids and asparagines in specific calcium-binding Epidermal Growth Factor (cbEGF) domains. Biologically, ASPH is involved in trophoblast invasion of the uterine wall and is expressed in the endoderm of developing embryos although expression in healthy adult tissue is extremely limited [16]. ASPH has been demonstrated to activate the Notch signalling pathway both in vitro and in vivo [16]. ASPH inhibitors have been rationally designed and synthesized, and demonstrate predicted activities in vitro [17], including suppression of migration, invasion, and activation of NOTCH pathway related proteins. The Notch signalling pathway has been identified as a key pathway in cancer [18]. In vivo proof-of-principle experiments demonstrate significant suppression of tumour growth at 1 mg/kg [19].

**Figure 1:**
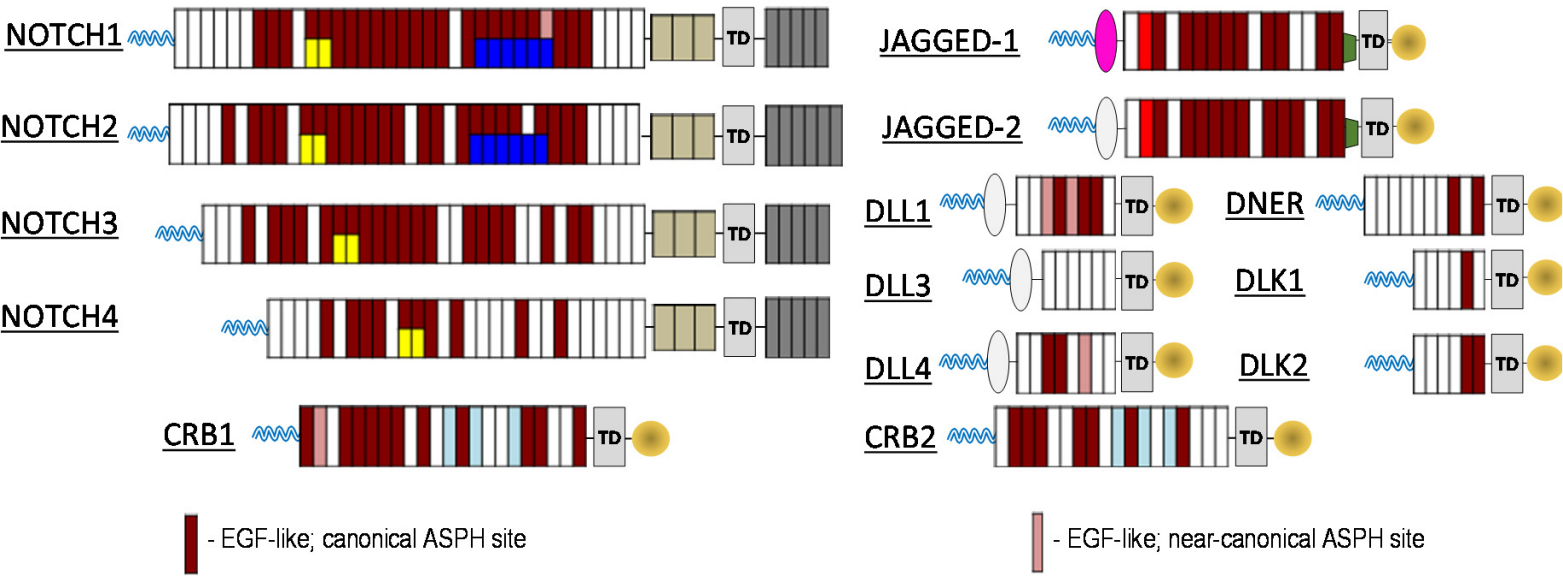
Presence of multiple canonical Aspartyl(asparaginyl)-β-hydroxylase (ASPH) hydroxylation sites in all cbEGF containing NOTCH receptors, ligands (JAGGED-1/2) and modulators such as Crumbs human homologs.

To investigate the relationship between ASPH and the Notch signalling pathway, computational prediction of ASPH substrates was performed utilizing the known ASPH hydroxylation recognition sequence. Both canonical as well as near canonical recognition sequences were evaluated. As illustrated in Figure 1, nearly all Notch receptors contain potential ASPH hydroxylation sites, as do known Notch ligands Jagged-1/2, DLL1, DLL4, DNER, DLK1/2. In addition, known Notch pathway modulating proteins CRB1/2 also contain potential ASPH hydroxylation sites. The results of these computational predictions enable the creation of new paradigm for cancer therapeutics.

**Figure 2:**
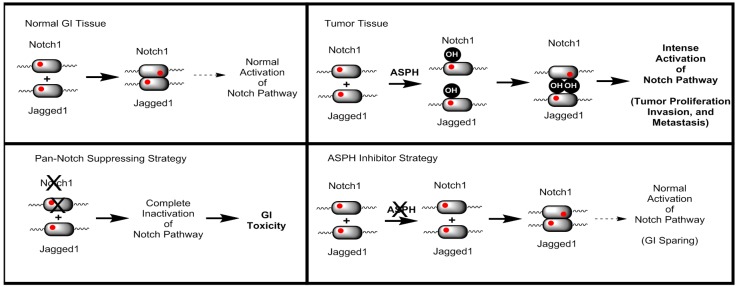
Illustration of NOTCH signalling for normal GI, tumour tissue, pan-notch suppressing strategy (such as γ-secretase inhibitor), and anti-ASPH GI sparing activity.

Previous strategies for targeting the Notch signalling pathway, such as γ-secretase inhibitors that prevent the proteolytic processing of Notch family receptors [20], suffer from severe, dose limiting gastro-intestinal (GI) toxicity, complicating the clinical potential of this class of agent [21]. However, ASPH inhibitors do not demonstrate GI toxicity even when administered IP at a dose of 50 mg/kg every other day. To rationalize this observation, a model for ASPH modulation of the Notch signalling pathway has been developed as illustrated in Figure 2.

Briefly, normal GI tissue requires a basal level of normal Notch pathway activation. Tumour tissue expresses ASPH, resulting in hydroxylated Notch receptors, ligands, and perhaps even modulators. As a consequence, there is a dramatic increase in Notch signalling resulting in intense activation. A Pan-Notch suppressing strategy, such as gamma-secretase inhibitors, suppresses all Notch activation, including the basal level of activation required for normal GI function. However, a specific ASPH inhibitor suppresses the tumour specific Notch pathway activation, while sparing the normal GI Notch signalling.

## 4. Design, Synthesis, and Anti-Cancer Activity of Tetrandrine Derivatives

PanW.LiuY.HuS.LanJ.

Natural products remain the most important source of pharmacological treatments nowadays. Based on statistics, more than 50% of drugs come from natural products, either natural origins or derivatives of natural origins [22,23,24]. One anticancer drug, paclitaxel, has saved or prolonged survival time since it was commercially introduced into the clinic, and has also attracted many research groups with the challenge of total synthesis and synthetic process [25,26]. Numerous similar compounds such as albumin-bound paclitaxel, docetaxel and taxanes are being developed for clinical use [27,28,29,30]. Many compounds, especially including natural products, have been studied with the rise of new mechanisms of cell death.

Alkaloid or alkaloid-containing plants play an important role in medicine for anticancer, antiarrhythmic, antihypertensive, muscle relaxant and so on [31,32]. Tetrandrine is a typical bisbenzylisoquinoline alkaloid which raised great attention with its bio-activities such as anti-cancer, anti-inflammatory, immunologic and antiallergenic effects since it has been isolated from a Chinese medicinal plant, *Stephania tetrandra* S Moore [33,34,35,36].

**Scheme 1:**
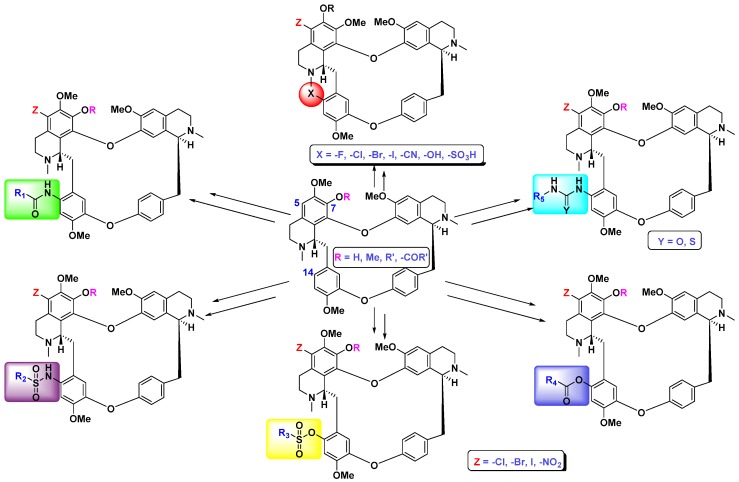
Design and synthesis of investigated alkaloid analogues.

Our group previously discovered that the analogues of tetrandrine and fangchinoline showed potent anticancer activities on different cancer cell lines. As the research of fangchinoline moved along, we found that fangchinoline could induce human hepatocellular carcinoma cells (HCC) death through an autophagic mechanism by inducing translocation of p53 and activating AMPK, which is totally different of tetrandrine [37]. Based on those findings, the derivatization of these two novel natural products has proceeded, as illustrated in Scheme 1. Over 300 derivatives have been synthesized based on this family of novel natural products and some of them exhibited much stronger anticancer activities than the prototype compounds. From these derivatives, the thiourea compound **LJJ-104** exhibited strong inhibitory effect on HCC cells with IC_50_ values of 1.06 μM, which is 15.8-fold lower than that of the starting material tetrandrine and 30.3-folds than that of the positive control sorafenib. The mechanism study showed that **LJJ-104** was capable of initiating endoplasmic reticulum stress-associated apoptotic cell death, and activation of JNK and caspase pathways were probably involved. A derivative of fangchinoline, **ZG-01**, showed better anti-HCC activity and the mechanistic study revealed it might induce autophagic cell death as well as HDAC inhibition and anti-CSC activity. These results shed light for new anti-cancer drug development from the two lead natural compounds, tetrandrine and fangchinoline.

## 5. *En Route* to New Anticancer Agents Based on Alkaloid Luotonin A

HaiderN.

**Figure 3:**
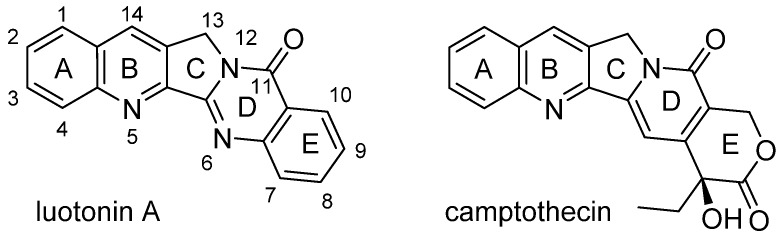
Structures of luotonin A and camptothecin (CPT).

Luotonin A is a pentacyclic alkaloid that was first isolated in the late 1990s from the Asian plant, *Peganum nigellastrum* Bunge (*Zygophyllaceae*) [38]. It has a pyrroloquinazolinoquinoline skeleton and thus shares some basic structural features with camptothecin (CPT, Figure 3). Camptothecin is well-known because of its pronounced antitumour activity [39]. The latter is based on a stabilising effect towards the binary complex formed between DNA and the enzyme, topoisomerase I, as an essential step for releasing the supercoiled structure of the nucleic acid, and this stabilisation effectively prevents DNA replication. While CPT itself has unfavourable pharmacokinetic properties, some of its derivatives, such as topotecan and irinotecan, have made their way into clinical use as anticancer agents for the chemotherapy of certain malignancies [39]. However, these camptothecin derivatives not only cause the typical side effects of most cytotoxic agents, but also suffer from specific adverse effects like bladder toxicity [40]. This problem arises from the labile hydroxylactone structure of CPT-like compounds, which first leads to an undesired early inactivation of the drug by lactone-ring opening at the physiological blood pH and later (during excretion) to another undesired structural change, namely re-formation of the lactone in the acidic environment of the urinary bladder. For that reason (together with synthesis-related aspects), luotonin A has received increasing interest as an alternative lead structure for the development of therapeutically useful topoisomerase I poisons, as it was found to exhibit a similar biological activity as CPT, albeit with considerably lower potency [41]. As shown in Figure 3, luotonin A does not possess the labile lactone structure and, moreover, lacks the chiral centre of CPT.

**Scheme 2:**
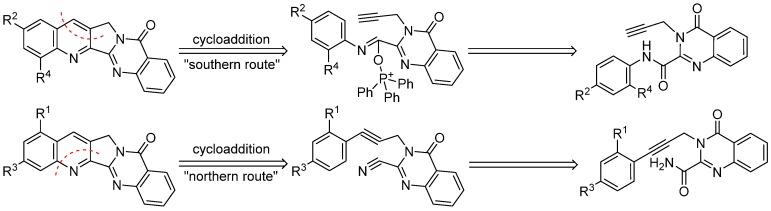
Retrosynthetic representation of two orthogonal synthetic routes to A-ring-substituted derivatives of luotonin A (for clarity, some bond angles in intermediates are drawn incorrectly).

Thus, in recent years quite a number of publications have appeared that describe various total syntheses of luotonin A as well as some structural modifications of this alkaloid. A comprehensive 2011 review article [41] summarises this development. Some of these synthetic pathways were used for the preparation of derivatives bearing various substituents mainly at rings A, B and E of the quinolino[2′,3′:3,4]pyrrolo[2,1-*b*]quinazoline system. In the Haider research group, two complementary synthetic strategies have been employed that permit the selective placement of different functionalities at positions 1, 2, 3, and 4 of the pentacyclic skeleton [42,43]: the “southern route” to 2- or 4-substituted derivatives makes use of a modified cycloaddition strategy that was first described by Zhou et al. [44] for the total synthesis of camptothecin and luotonin A; the “northern route” to 1- or 3-substituted congeners also utilizes an intramolecular [4 + 2] cycloaddition reaction as the key step, but features a different arrangement of the diene and dienophile components (Scheme 2).

A library of differently substituted derivatives of the alkaloid as well as some heterocyclic analogues could be prepared by these two routes, but both pathways were also found to have specific limitations. For instance, *N*-pyridyl residues are not tolerated as anilide-type synthons in the “southern route” [42], probably because of preferential attack at the pyridine nitrogen (and not at the amide oxygen) by the electrophilic phosphorus in bis(triphenyl)oxodiphosphonium triflate (Hendrickson’s reagent) that is used to promote the final cycloaddition step. A similar regioselectivity issue was recently observed with anilides bearing an ester group or a cyano group in *ortho* position, whereas the *para*-substituted counterparts smoothly undergo the cycloaddition reaction in the expected way [45].

**Scheme 3:**
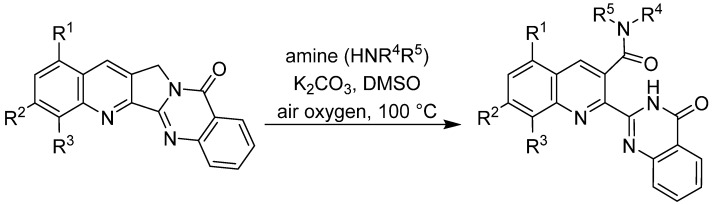
Oxidative opening of ring C on reaction of luotonin A or derivatives with amines/K_2_CO_3_ in DMSO in presence of air oxygen.

Several of the A-ring-modified Luotonin A derivatives that have been synthesised via these two routes exhibit in vitro growth inhibitory activity towards selected human tumour cell lines with EC_50_ values in the low micromolar range but so far, no candidate with sub-micromolar activity could be obtained. In an attempt to further modify the lead structure, nucleophilic displacement of a 4-fluoro substituent with primary and secondary amines was investigated [46]. Here, an unexpected oxidative C-ring opening reaction was observed that leads to carboxamides of the general structure shown in Scheme 3. Interestingly, some of these novel compounds show moderate cytotoxic activity.

## 6. Targeting Oncogenic Mutant TP53—One Step Ahead

MusiolR.

Protein p53, encoded by TP53 tumour suppressor gene is one of the most important nodes in cell’s life and death cycle. It is responsible for response to major stress factors such as electromagnetic radiation, oxidative stress, DNA damage or metabolic disturbances [47]. In healthy cells p53 is suppressed by endonucleases Mdm2/MdmX through a ubiquitination trigger for proteasomal degradation. Stress factor phosphorylation stops the Mdm2 protein and activates p53 which triggers pathways leading to cell cycle arrest, DNA repair or in case of fatal damage; induction of apoptosis [47,48,49]. For this reason, p53 has potent control activity in cell development. On the other hand, Tp53 is also one of the most frequently mutated genes in cancers [50]. It can be found in roughly half of all cancer cases with high prevalence in breast, lung and colon cancers. Significantly, the prognosis is often poor in cases with diagnosed p53 mutation [51]. A primary challenge concerning TP53 mutation is the extremely high variability of the type and position resulting in vast landscape of mutants. Although the majority of the mutants lose their suppressor functionality, there are also mutants that acquire even more oncogenic strength—the so-called gain-of-function mutations [52]. The function and prevalence in cancers make p53 a good drug target for anticancer therapy [53]. Various drugs suppressing mutant p53 have been described, however only PRIMA-1 (APR-246) has reached clinical trials so far [54]. Unfortunately, most p53 targeting drugs seem to be predominantly selective for specific mutants and they tend to remain inactive in cancers with deletion of *Tp53* (p53 *null*).

**Scheme 4:**
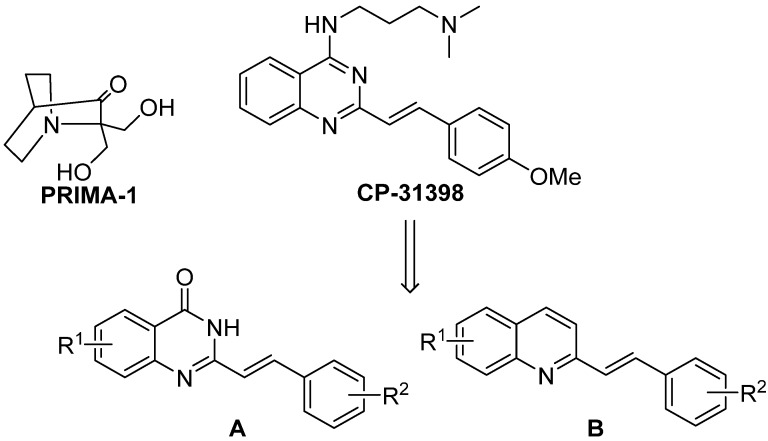
Design of CP-31398 analogues as anticancer agents—styrylquinazolinones (**A**) and styrylquinolines (**B**).

During our research, we concentrated on the known styrylquinazoline derivative CP-31398 (see Scheme 4) with p53 reactivating activity. This compound interacts with the DNA-binding domain of p53 [55,56]. It is also able to increase synthesis of this protein in mutants and in wild type cells. In p53 deficient cells, this compound is also capable of inducing apoptosis through an independent pathway; however, the activity is greatly diminished. As presented in Table 1, against p53 null mutants, the weak anticancer activity of CP31398 results in an unacceptably low therapeutic index when compared to normal human fibroblasts (GM07492). Given our strong experience in quinoline derivatives with antifungal [57,58], antimicrobial [59,60,61,62] and antiretroviral [63,64,65] activity, we decided to pursue our interest in quinolines targeting p53 deficient cells in the search for novel anticancer agents. Quinoline [66,67,68,69,70,71] and quinazoline [72] derivatives synthesized in our group showed strong anticancer activity. Novel compounds designed in this study appeared to be several times more effective against p53^−/−^ than normal cells. Interestingly, this potency seems to be related with the intercalating properties of styrylquinolines.

In conclusion, mutations in TP53 could be considered an Achilles heel of cancer. Although the absence of suppressor proteins opens the way for proliferation and drug resistance, it also makes the cells more vulnerable to DNA damage. Low concentrations of intercalating agents lead to small DNA damage. This may be detected and repaired under control of properly functioning p53 protein, while p53 null mutants suffer from accumulation of these injuries and die much earlier.

## 7. Highly Anticancer Active Copper(II) Complexes Involving Natural and Nature-Inspired *O,O′*-Donor Ligands

VancoJ.

Secondary metabolites of higher plants, such as flavonoids, 2′-hydroxychalcones or β-diketones, show plenty of important biological activities as well as represent suitable *O,O′*-chelating ligands for the preparation of biologically active transition metal complexes.

The recent trends in the design and development of anticancer active coordination compounds involve several diverse strategies. The efforts to design innovative drugs based on traditional platinum-based clinically used drugs, such as cisplatin, often fails due to unfavourable benefit/risk ratio and such new potential drugs already represent only about 20% of all metal-based cancer therapeutics in ongoing clinical trials [74]. It seems likely that one of the viable routes towards new and more active cancer therapeutics may be represented by the development of non-platinum coordination compounds. One of the interesting and prospective groups of non-platinum based biologically-active compounds is represented by heteroleptic copper complexes. Recently, a member of a group of mixed-ligand copper(II) complexes, known under the name of Casiopeínas^®^ [75], involving the combination of heterocyclic diimine *N,N′*-donor ligands and simple additional *N,O*- or *O,O′*-ligands, made it as far as to Phase 1 clinical trials as a prospective anticancer agent [76].

In an effort to prepare new and more active anticancer mixed-ligand copper(II) complexes, involving the combination of diimine *N,N′*-donor ligands and additional *O,O′*-ligands, we decided to utilize the nature-inspired *O,O′*-ligands from the groups of flavonoids, 2′-hydroxychalcones [77] and their synthetically modified analogues. As the results of our endeavours realized to date, three generations of highly biologically interesting compounds were prepared and studied by a series of chemical, physical and biological tests used to describe their structure, physical, chemical properties and affinity towards biological targets which enabled us to uncover some mechanisms responsible for their potent antitumor effects.

**Figure 4:**
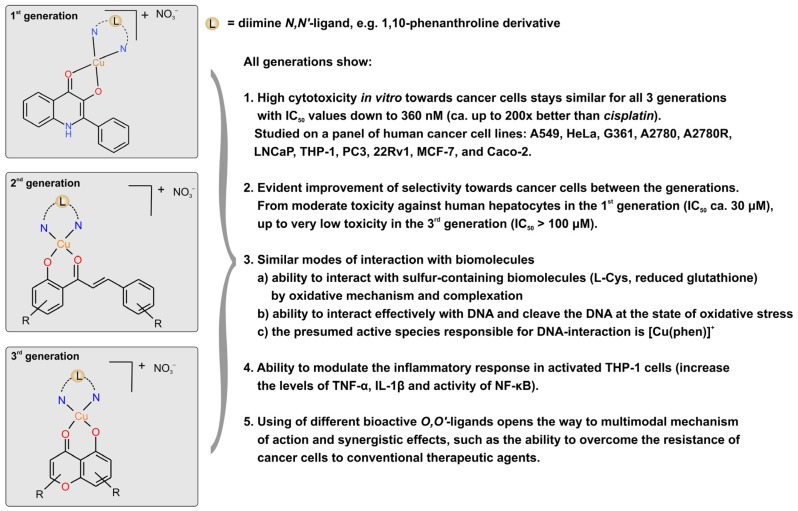
The overview of structural types of studied generations of mixed-ligand copper(II) complexes and their biological activities.

The first generation of the mixed-ligand copper(II) complexes (see Figure 4) involved a combination of 1,10-phenanthroline derivatives and 1-azaflavone-3-ol ones [78,79]. The most active compounds showed significant cytotoxicity against the panel of human cancer cell lines (A549, HeLa, G361, A2780, A2780R, LNCaP, THP-1), with IC_50_ values in the range of 360 nM–3.9 μM, and moderate toxicity towards primary culture of human hepatocytes, with IC_50_ values up to 20 μM.

The second generation of the copper(II) complexes (see Figure 4) involved a combination of 1,10-phenanthroline derivatives and 2′-hydroxychalcone derivatives. The most active compounds showed significant cytotoxicity against the panel of human cancer cell lines (A549, HeLa, G361, HOS, A2780, A2780R, 22Rv1), with IC_50_ values in the range of 1.1 μM–9.3 μM, and lower toxicity towards primary culture of human hepatocytes, with IC_50_ values up to 64 μM [80].

The third generation of the copper(II) complexes (see Figure 4) involved a combination of 1,10-phenanthroline derivatives and flavonoid derivatives, intrinsically possessing a moderate anticancer activity. The most active compounds showed significant cytotoxicity against the panel of human cancer cell lines (A549, MCF-7, HOS, A2780, A2780R, Caco-2), with IC_50_ values in the range of 600 nM–5.9 μM, and very low toxicity towards primary culture of human hepatocytes, with IC_50_ values up to more than 100 μM (limited by the solubility of the compound).

All three generations of complexes showed the ability to effectively interact with DNA by intercalative mechanism and act as chemical nucleases in the state of oxidative stress. Moreover, the compounds were able to interact with the sulphur-containing biomolecules l-cysteine and reduced glutathione at physiologically relevant conditions by both an oxidative mechanism and by formation of binary complexes.

The synergistic anticancer effect of *O,O′*-ligands in the compounds from the third generation led to the more pronounced specificity and selectivity of their action towards the various cancer cell lines and richer spectrum of cellular and molecular effects as compared to previous generations of compounds. These compounds therefore represent very promising agents for further preclinical testing.

## 8. Non-Canonical Cell Death Mechanisms as a Pharmacological Target

DiederichM.JiS.

Over recent years, drug discovery and development essentially focused on compounds whether natural or synthetic able to trigger apoptotic cell death. Essential readouts were the coordinated activation of caspase cascades culminating in PARP-1 cleavage, nuclear fragmentation and pyknosis followed by a complete elimination of apoptotic bodies formed by this process. Accordingly, drug development focused on compounds able to activate both extrinsic or intrinsic cell death cascades or synergistic activation of both. In recent years, potent activators of mitochondrial cell death mechanisms including clinically used Bcl-2 inhibitors entered clinical trials and were FDA approved. Venetoclax (Venclexa) is an excellent example of such a synthetic compound targeting Bcl-2 with high specificity alone or combined to other pharmacologically active compounds [81].

Besides apoptotic cell death, other modalities of cellular demise became progressively better understood and entered the theatre of potential drug development targets. Controlled necrosis was no longer considered as a catastrophic potentially harmful mechanism of cell demise but researchers like Vandenabeele [82] started to investigate closer such mechanisms and derive programmed aspects in line with the basic principles of programmed cell death coined by Lockshin in the 1960s [83].

Various mechanisms of controlled necrosis were identified recently [84]: Necroptosis is intensively studied programmed necrosis and RIPK is an initial key regulator to foam necrosome to disrupt plasma membrane. Besides, a dysregulation of reactive oxygen species (ROS) and Ca^2+^ homeostasis largely leads to cell demise. Parthanatos occurs by hyperactivation of PARPs, typically PARP-1, which is leading to depletion of NAD^+^ and ATP. PARP-1 is a DNA repair protein and activated by ultraviolet light, ROS, alkylating agents and Ca^2+^ signalling pathways. 3-aminobenzamide (3-AB) is a typical pharmacological inhibitor for this cell death modality. It was recently reported that inhibition of the xc-cystine/glutamate antiporter induced regulated necrosis by facilitating import of cystine and export of glutamate. This new regulated necrosis was named oxytosis and triggered lysosomal membrane permeabilization (LMP) through calpain activation. Such pathways are to become novel cell death pathways with identifiable pharmacological targets to be exploited for future cancer treatments. Interestingly these “novel” cell death pathways most frequently lead to potent danger associated molecular pattern (DAMP) exposure, described by Kroemer as particularly immunogenic as DAMP release characterized immunogenic cell death or ICD [85].

In conclusion, non-canonical cell death induction culminating in ICD could thus contribute to the development of anticancer treatments with both a cytotoxic component and an outcome triggering the essential immune response of patients.

## 9. Structural Modifications of Pyrazinamide and Pyrazinoic Acid—Rational Design towards New Potential Antituberculars

ZitkoJ.

### 9.1. Introduction

According to the latest WHO Global Tuberculosis Report, estimated 10.4 million people worldwide developed active tuberculosis (TB) in 2015 [86]. In 2015, TB was the causative agent of 1.4 million deaths, including 0.4 million deaths in people with HIV/TB co-infection [86]. Since 2015, TB is the leading cause of death among all infectious diseases. The wide distribution of drug-resistant TB strains is threatening the TB control policy.

The first-line antitubercular drug pyrazinamide (PZA) has been in clinical use since 1950’s [87]. Despite its firm position in combination therapy of TB, the mechanism of action (MoA) of PZA is not yet fully understood. For more than 50 years, PZA was supposed to act by non-specific MoA based on acid-base properties of its active metabolite - pyrazinoic acid (POA), produced by intracellular hydrolysis of PZA catalyzed by mycobacterial pyrazinamidase/nicotinamidase (PncA). Intracellular accumulation of POA leads to acidification of cytoplasm, disruption of membrane potentials and interferes with processes dependent on proton gradient [88]. This theory was the most-widely accepted MoA of PZA for a long time. In 2015, this non-specific MoA was challenged by Peterson et al. [89], who directly measured the intracellular pH of mycobacterial cells cultivated with PZA or POA. They observed only minor, probably biologically irrelevant, acidification after prolonged cultivation with PZA/POA at concentration 10-fold higher than reported minimum inhibitory concentrations (MIC). At concentration of 1× MIC, no acidification was observed. Similarly, no significant disruption of membrane potential was observed at biologically relevant concentrations of PZA/POA.

Since 2000, four different subcellular targets of PZA and/or POA have been reported. Actually, the discovery of three of them happened in the last six years (since 2011). The level of proof of individual proposed targets varies, as well as their usability in rational structure-based drug design (SBDD) efforts.

### 9.2. Inhibition of Fatty Acid Synthase Type I

Mycobacterial Fatty Acid Synthase type I (FAS I) produces C_16_ and C_26_ fatty acids. Both C_16_ and C_26_ acyl derivatives are then utilized by FAS II system for the synthesis of mycolic acids, specific and unusually long fatty acids typical for mycobacteria. Fatty acid synthesis is a classical target of antiTB drugs, as the first-line isoniazid inhibits (after activation) the enoyl-(acyl carrier protein) reductase (InhA), which belongs to the FAS II system. Inhibition of FAS I as a MoA was proved for PZA, POA, and simple derivatives such as 5-Cl-PZA or *n-*alkyl esters of POA. The inhibition of FAS I was proved by means of enzyme inhibition in whole cells [90,91], inhibition of isolated enzyme [91,92], and gene resistance studies [93]. The first studies reporting FAS I inhibition as potential MoA of PZA occurred in 2000 [93], but it was not until 2011 when light was shed on the molecular mechanism of FAS I inhibition by PZA and related compounds. Sayahi et al. showed by Saturation Transfer Difference NMR experiments on isolated enzyme that PZA [94] and its derivatives [95] are competitive inhibitors of FAS I, competing for the binding site of NADPH cofactor. Out of six catalytic domains of FAS I, only two of them utilize NADPH, namely ketoacyl reductase domain and enoyl reductase domain. Therefore, according to current knowledge, PZA derivatives interact on molecular level with one or both of these domains of FAS I. FAS I is a large homohexameric protein (2 MDa), therefore efforts to obtain crystals of sufficient quality for X-ray diffraction studies we not successful so far. The only 3D structure of mycobacterial FAS I is a model based on cryo-electron microscopy [96], but this structure does not have sufficient resolution for direct usage in SBDD methods.

### 9.3. Inhibition of Trans-Translation

**Figure 5:**
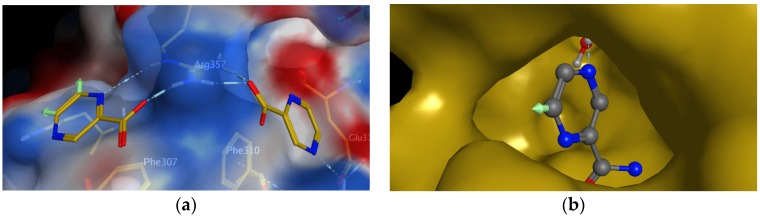
Exit vectors indicated by green arrows designate possible structural modifications of pyrazinamide (PZA) / pyrazinoic acid (POA): (**a**) POA bound to surface of ribosomal protein S1 (pdb: 4NNI) [98]; (**b**) PZA docked into catalytic site of pyrazinamidase PncA (pdb: 3PL1).

*Trans*-translation is a process of rescuing ribosomes stalled during faulty translation of mRNA. As published in 2011, Shi et al. was fishing for the specific target of POA in a smart way [97]. They covalently bound 5-hydroxypyrazinoic acid (5-OH-POA) to polymer stationary phase. This POA bait was then used to fish for the target protein in the pool of mycobacterial lysate (affinity chromatography). The ‘fish’ with the highest affinity to the POA bait was the ribosomal protein S1 (RpsA). Isothermal titration calorimetry proved POA binding to RpsA. The crucial step of trans-translation is the RpsA mediated attachment of tmRNA to the translating complex. It was shown that POA inhibits tmRNA-RpsA association in a dose-dependent manner [97]. As another level of indirect proof of this MoA, it was found that some PZA-resistant strains of *M. tuberculosis* possess mutations in *rpsA* gene [97]. In 2015, Yang et al. deposited an X-ray resolved structure of POA-RpsA complex (pdb: 4NNI) [98]. Interestingly, they observed that two molecules of POA are bound to the surface of RpsA. POA binds to the close vicinity of residues that are known to interact with tmRNA, which is in agreement with the proposed molecular MoA. As visible in Figure 5a (indicated by green arrows), one molecule of POA can be substituted on C-5 or C-6 of the pyrazine nucleus. As authors logically suggested, these positions should be substituted with lipophilic substituents to increase the penetration of rather hydrophilic POA through the mycobacterial cell wall [98].

### 9.4. Other Specific Targets for PZA

In 2014, it was shown that POA inhibits l-aspartate α-decarboxylase (PanD) of *M. tuberculosis* [99]. PanD is an important enzyme in pantothenate synthesis. The molecular mechanism of inhibition is not known, although there were efforts to find the binding site of POA in PanD by molecular docking combined with molecular dynamics [100]. 3D structure of PanD is available as pdb: 2C45. Again in 2014, PZA was recognized as an inhibitor of quinolinic acid phosphoribosyl transferase (QAPRTase) of *M. tuberculosis* [101]. QAPRTase catalyzes the synthesis of nicotinic acid mononucleotide from quinolinic acid and 5-phosphoribosyl-1-pyrophosphate, in a biosynthetic pathway leading to NADH. Molecular docking suggested that PZA can compete for the binding site with the natural substrate – quinolinic acid [101]. 3D structure of QAPRTase is available as pdb: 1QPO.

### 9.5. Implications for Structure-Based Drug Design

In recent years, the perception of PZA and its metabolite POA has changed from a non-specific cytosol acidifier to a multi-target inhibitor of specific mycobacterial enzymes and processes. Current knowledge of specific targets and availability of their 3D crystallographic structures is a starting point for rational SBDD to prepare PZA/POA derivatives with improved pharmacodynamical/pharmacokinetic properties. Often, PZA is a prodrug of POA, which itself is the inhibitor of specific targets as in the case of RpsA and PanD. FAS I is inhibited by both PZA and POA, as well as by some simple structural derivatives. Interestingly, QAPRTase is inhibited by PZA but not POA.

As an example of rational design of PZA/POA derivatives, we have designed and prepared a series of 5- or 6-substituted PZA derivatives, using lipophilic substituents such as alkyl, alkylamino [102] or alkylamido (C_3_-C_8_). The rationale for substitution at C-5 or C-6 on the pyrazine core is given by interactions of POA-RpsA (Figure 5a). To address the question whether 5- or 6-substituted PZA can still be hydrolyzed by PncA to the corresponding substituted POA, a molecular docking study was performed. Preliminary results show that at least some 6-substituted PZA derivatives (but probably not 5-substituted PZA derivatives) could be substrates of PncA (Figure 5b).

## 10. Natural Products Regulating Macrophage Cholesterol Efflux

AtanasovA.G.WangD.

### 10.1. Introduction

Macrophages scavenge the oxidized low-density lipoprotein (ox-LDL) to become cholesterol-laden “macrophage foam cells” [103]. Macrophage foam cell formation and accumulation in the subendothelial area of the arterial wall is the hallmark of atherosclerosis [103]. Recent clinical reports indicated that enhancement of macrophage cholesterol efflux is a promising strategy for the prevention and treatment of atherosclerosis [104]. Natural products represent an important pool for the identification of novel drug leads, which is supported by the fact that natural products have become effective drugs in a wide variety of therapeutic indications, and are broadly studied in the context of cardiovascular disease therapy in particular [105,106,107,108]. Therefore, lots of studies by us and others have addressed natural products that modulate macrophage cholesterol efflux. In this part of the manuscript, we summarize interesting studies published in recent years in this research area.

### 10.2. Flavonoids and Polyphenols

Flavonoids and polyphenols are an important source for seeking natural products with macrophage cholesterol efflux enhancing capacity. Alpinetin, a natural flavonoid abundantly present in the ginger family, exhibited positive effects on cholesterol efflux and inhibited ox-LDL-induced lipid accumulation in ox-LDL-loaded THP-1 macrophages and human monocyte-derived macrophages (HMDM), which might be through the peroxisome proliferator-activated receptor γ (PPARγ)/liver X receptor-alpha (LXRα)/ATP-binding cassette transporter A1 (ABCA1)/ABCG1 pathway [109]. Baicalin, a major flavonoid of *Scutellaria baicalensis*, increased cholesterol efflux from THP-1 macrophages possibly through the PPARγ-LXRα-ABCA1/ABCG1 pathway [110]. In vivo, treatment with baicalin significantly decreased atherosclerotic lesion sizes and lipid accumulation in atherosclerosis rabbit carotid arteries [110]. Another study shows that baicalin induced cholesterol efflux from THP-1 macrophages via the PPARγ/LXRα/SR-BI pathway. Chrysin, a natural flavonoid, promoted cholesterol efflux from RAW264.7 macrophages, which may be related to up-regulation of the classical PPARγ-LXRα-ABCA1/ABCG1 pathway [111]. Hesperetin, a citrus flavonoid, promoted apoA-I-mediated cholesterol efflux from macrophages by increasing ABCA1 expression through the activation of LXRα and PPARγ [112]. Quercetin, a flavonoid, increased cholesterol efflux from THP-1 derived macrophages through activating PPARγ-LXRα-ABCA1 pathway [113]. Another study indicated that quercetin enhanced cholesterol efflux, which was associated with an increase in ABCA1 mRNA and protein expression through p38-LXRα-dependent pathway [114]. Silymarin is a hepatoprotective mixture of flavonolignans and flavonoids extracted from the seeds of milk thistle (*Silybum marianum* L. Gaertn). Its major constituents, including isosilybin A, silybin B, silychristin and isosilychristin, promoted cholesterol efflux from THP-1 macrophages via upregulation of the ABCA1 protein levels [115].

Polyphenols beyond flavonoids are also the important source for seeking natural products with macrophage cholesterol efflux enhancing capacity. Consumption of extra virgin olive oil-enriched with green tea polyphenols stimulated cholesterol efflux rate from mouse peritoneal macrophages (MPM) [116]. In vivo experiments indicated that consumption of this extract also reduced atherosclerotic lesion size of mice significantly [116]. Unfortunately, the authors could not conclude that the antiatherogenicity of the extract is strictly due to its effect on cholesterol efflux, since the extract also increased high-density lipoprotein (HDL) cholesterol levels in serum. Paeonol, a phenolic component purified from *Paeonia suffruticosa* (*Cortex Moutan*), enhanced cholesterol efflux from J774.A1 macrophages by LXRα-ABCA1-dependent pathway [117]. In vivo study also indicated that paeonol increased protein expression of ABCA1 in apolipoprotein E (apoE) deficient (apoE^−/−^) mice [117]. Pomegranate peel polyphenols promoted apoA-I-mediated macrophage cholesterol efflux from RAW264.7 macrophages by up-regulating ABCA1 and LXRα at the mRNA and protein levels, with independence on ABCG1 and PPARγ [118].

### 10.3. Carotene and Its Derivatives

9-*cis*-Retinoic acid, which is synthesized primarily intracellularly from retinaldehyde that can be produced from β-carotene, induced macrophage cholesterol efflux via the LXRα-dependent up-regulation of ABCA1 and ABCG1 [17]. 9-*cis*-Retinoic acid also reduced the atherosclerotic plaque area in the aortic sinus of apoE^−/−^ mice [119]. 9-*cis*-β-carotene, a precursor for 9-*cis*-Retinoic-acid, as well as all-*trans*-β-carotene significantly increased cholesterol efflux from RAW264.7 macrophages, which might be ascribed to transcriptional induction of ABCA1, ABCG1, and apoE [120]. Astaxanthin, one of the naturally occurring carotenoids, increased ABCA1/G1 expression, thereby enhancing apoA-I/HDL-mediated cholesterol efflux from RAW264.7 cells in a LXR-independent manner [121].

### 10.4. Fatty Acids

Dietary consumption of fatty acids has been reported to be related to atherosclerosis [122]. 13-hydroxy linoleic acid, a natural PPAR agonist, induced cholesterol efflux from RAW264.7 macrophages via the PPARα/β-LXRα-ABCA1/SR-BI-pathway [122]. Elaidic acid, the most abundant *trans*-fatty acid in partially hydrogenated vegetable oil, slightly increased ABCA1-mediated cholesterol efflux, while oleic acid led to decreased ABCA1-mediated efflux [123]. Elaidic acid was shown to stabilize macrophage ABCA1 protein levels in comparison to that of its *cis*-fatty acid isomer, oleic acid, which accelerated ABCA1 protein degradation [123]. Linoleic acid (18:2), an unsaturated fatty acid, attenuated HDL-mediated cholesterol efflux from murine bone marrow-derived macrophages (BMDM) through down regulation of ABCA1 mRNA and protein levels but not through changes in LXRα or sterol receptor element binding protein (SREBP)-1 expression.

### 10.5. Other Compounds

Curcumin from the rhizomes of *Curcuma longa* affected the cholesterol efflux from adipocytes by regulating the PPAR-LXR-ABCA1 pathway [124]. It is also demonstrated that curcumin enhanced cholesterol efflux from macrophage via suppressing the JNK pathway and activating the LXR-ABCA1/SR-BI pathway [125]. Furthermore, curcumin enhanced cholesterol efflux by upregulating ABCA1 expression through activating AMP-activated protein kinase (AMPK)-SIRT1-LXRα signalling in THP-1 macrophage-derived foam cells [126].

Falcarindiol and other polyacetylenes have been demonstrated to act as agonists of PPARγ, an important transcriptional regulator of cholesterol efflux [127,128]. Interestingly, falcarindiol increased ABCA1 protein level and macrophage cholesterol efflux not just by activating PPARγ and following promotion of ABCA1 gene expression, but also by a second complementary mechanism involving inhibition of ABCA1 protein degradation [129].

Danshensu, the major water-soluble component extracted from *Salvia miltiorrhiza* Bunge (Danshen), promoted cholesterol efflux from RAW264.7 macrophages by the upregulation of the cellular cholesterol exporters ABCA1 and ABCG1 [130]. Diosgenin, a structural analogue of cholesterol from Danshen, enhanced ABCA1-dependent cholesterol efflux and inhibited aortic atherosclerosis progression by suppressing macrophage miR-19b expression in human THP-1 macrophages and MPMs [131]. Salvianolic acid B, also known as lithospermic acid B or tanshinoate B isolated from Danshen, promoted cholesterol efflux from THP-1 macrophages through the PPARγ/LXRα/ABCA1 pathway [132].

Arctigenin, a bioactive component from *Arctium lappa*, promoted cholesterol efflux from ox-LDL-loaded THP-1 macrophages through upregulation of ABCA1, ABCG1 and apoE, which was dependent on the enhanced expression of PPARγ and LXRα [133]. Citrulline, which is unusually abundant in watermelon (*Citrullus vulgaris*), increased ABCA1 and ABCG1 mRNA and protein levels in THP-1 macrophages and monocyte-derived macrophages, translating into enhanced apoA-I- and HDL-mediated cholesterol efflux [134]. Emodin, an anthraquinone derivative isolated from the roots of *Rheum palmatum*, enhanced cholesterol efflux by activating PPARγ in ox-LDL-loaded THP1 macrophages [135]. Erythrodiol from olive oil promoted cholesterol efflux from THP-1-derived macrophages by stabilizing the ABCA1 protein [136]. Leoligin, a natural lignan found in Edelweiss (*Leontopodium nivale* ssp. *alpinum*), induced cholesterol efflux from THP-1-derived macrophages by upregulating ABCA1 and ABCG1 expression [137]. Piperine, the principle pungent constituent of the fruits of *Piper nigrum* L., promoted cholesterol efflux from THP-1-derived macrophages probably via interference with the calpain-mediated ABCA1 degradation pathway [138]. Puerarin, the effective component isolated from the Chinese herb *Pueraria lobate*, promoted ABCA1-mediated cholesterol efflux and decreased intracellular cholesterol levels through the pathway involving miR-7, STK11, and the AMPK-PPARγ-LXRα-ABCA1 cascade [139]. Spiromastixones 6 and 14, which are from a deep sea-derived fungus (*Spiromastix* sp. MCCC 3A00308), stimulated cholesterol efflux to apoA-I and HDL in RAW264.7 macrophages through upregulation of PPARγ and ABCA1/G1 [140]. Vitamin D, a group of fat-soluble secosteroids, stimulated cholesterol efflux through induction of CYP27A1 expression via a vitamin D receptor-dependent c-Jun *N*-terminal kinase (JNK) 1/2 signalling pathway and increase of 27-hydroxycholesterol levels, which induced LXR, ABCA1 and ABCG1 expression [141].

Sesamol and sesame (*Sesamum indicum*) oil enhance macrophage cholesterol efflux via up-regulation of PPARγ1 and LXRα transcriptional activity in a MAPK-dependent manner [142]. Sesamin, an active constituent of *Sesamum indicum*, enhanced cholesterol efflux from RAW264.7 macrophages probably through upregulation of the PPARγ-LXRα-ABCG1 pathway [143]. Another study suggested that sesamin improved macrophage cholesterol efflux through PPARγ1-LXRα and MAPK signalling [144]. Vascular smooth muscle cells (VSMC) also can form foam cells via accumulation of cholesterol. 17β-Estradiol promoted cholesterol efflux from VSMC and reduced VSMC-derived foam cell formation via LXRα-dependent upregulation of ABCA1 and ABCG1 [145]. Bilirubin, a strong antioxidant, decreased macrophage cholesterol efflux from cholesterol-loaded THP-1 macrophage foam cells, which was associated with decreased ABCA1 protein expression [146]. Nicotine, an important component in tobacco, suppressed cholesterol efflux from HMDM by inhibiting LXR pathway [147].

### 10.6. Conclusions

Natural products from traditional medicine and food represent an important pool for the identification of compounds with cholesterol efflux enhancing capacity. In this part of the manuscript, we summarize compounds with various kinds of chemical structure including flavonoids, polyphenols, and fatty acids, carotene and its derivatives, and lignin, etc., which could regulate cholesterol efflux from macrophages. The mechanisms of increased cholesterol efflux by these natural compounds include regulation of PPARγ-LXRα-ABCA1/ABCG1/SR-BI pathway, p38-LXRα-ABCA1 pathway, AMPK-SIRT1-LXRα-ABCA1 pathway, and AMPK-PPARγ-LXRα-ABCA1 pathway.

## 11. Trends in Design of Novel Ligands for Treatment of Neurodegenerative Diseases

AgbabaD.NikolicK.OljacicS.VucicevicJ.

### 11.1. Introduction

Pathophysiological mechanisms of neurodegenerative diseases (such as Alzheimer’s Disease (AD) and Parkinson’s Disease (PD)) are based mostly on very complex deregulation of cholinergic, adrenergic, dopaminergic, serotonergic, glutamatergic, and histaminergic neurotransmitter systems. Pathophysiology of AD also includes formation of the β-amyloid fibril deposits and β-amyloid oligomers. Current therapeutic strategies for the treatment of AD are based on acting on one drug target only and therefore show limited efficacy in modifying the disease. Since AD is a complex multi-factorial disease with various clinical symptoms, such as multiple cognitive deficits affecting memory, attention and language, development of an efficient therapy is very challenging [148]. The lack of efficacy of the single-target approach observed so far has led to the development of the multi-target drug design and polypharmacological drug discovery approach [149,150].

### 11.2. Design of Novel Ligands

**Figure 6:**
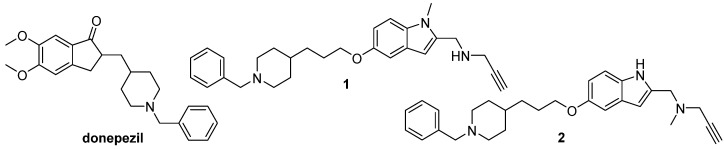
Chemical structure of donepezil and two new multi-target ligands against Alzheimer’s Disease (AD) [153,154].

Current trends in design and discovery of novel ligands for the treatment of neurodegenerative diseases are based on discovery of a set of drug targets for these diseases and on development of ligands with an optimal action on a few drug targets. Computational approaches such as data mining/repurposing, ligand/structure-based drug design, and in-silico screening are very successfully used for the design of the multi-target drugs with a desired activity profile. The in-silico methods are very useful in the discovery and optimization of novel ligands with an enhanced affinity to a set of the drug targets, as well as in optimization of physicochemical and pharmacokinetic properties of the multipotent drug candidates [151]. The ligand based virtual screening study [152,153] was successfully applied for lead optimization of the two donepezil hybrid compounds with the previously confirmed MAO-A, MAO-B, AChE, and BuChE inhibiting properties [154] (Figure 6). The pharmacophore analysis was performed to define the molecular determinant of these donepezil hybrids, to propose structural modifications that would increase inhibition of the enzymes, and to evaluate activities of the designed hybrids [152,153]. The main chemical diversities, pharmacophores and pharmacological profiles of the agents acting as a histamine H_3_R antagonist/inverse agonist and dual H_3_R antagonist/inverse agonist with an inhibiting effect on acetylcholine esterase, histamine *N*-methyltransferase, and the serotonin transporters were successfully developed in a few recent studies [155,156].

### 11.3. Conclusions

Neurodegenerative diseases such as AD and PD are multifactorial complex diseases affecting several related pathological pathways. Multi-target drug design is a promising approach in modern medicine for complex diseases. The new challenge is to define optimal combinations of the drug targets able to enhance the efficacy of the therapy.

## 12. Synthesis of Drugs Needs Synthesis of Knowledge: How is it with Stress, Anxiety and Depression?

JezovaD.

Identification of novel potential targets and gathering new knowledge belong to the necessary prerequisites of advanced drug development. In modern society, one of the most frequent causes of different pathological states is the exposure to intensive and chronic stress situations [157]. It is indisputable that chronic stress exposure significantly contributes to the development and course of psychiatric disorders. In spite of this, no anti-stress drug is in clinical use. The reason is that there is a strong evidence also of positive aspects of the stress response [158,159].

The synthesis of current knowledge clearly indicates that the synthesis of anti-stress drugs is not desirable. It is necessary to find new ways of decreasing the allostatic load by better coping with demanding stress situations occurring in the everyday life. New drugs are needed to improve the treatment of the negative consequences of stress exposure, such as anxiety and depression. We have identified a new target for future psychotropic drug development, namely the modulation of adrenocortical steroid aldosterone action in the brain. We have presented original data on anxiogenic and depressogenic action of aldosterone in animals [160] as well as changes in aldosterone release in patients with high trait anxiety and major depressive disorder [161,162].

Another new target for anti-depressant drug development is the β3-adrenergic receptor. The β3-adrenergic receptor is a frequently investigated target for pharmaceutical chemists and the derivatives of β3-adrenergic receptor agonists are being developed for their antioxidant, antimicrobial and other actions. A doctoral thesis on the synthesis and analysis of derivatives with potential action on β3-adrenergic receptors performed at the Department of Pharmaceutical Chemistry, Faculty of Pharmacy of the Comenius University in Bratislava [163,164] has motivated our research group to focus on the potential role of β3-adrenergic receptors in the stress response. In spite of the fact that catecholamines belong to the main stress hormones and they are acting via adrenergic receptors, no information has been available on possible changes of the β3-adrenergic receptors under stress conditions. We have provided the first evidence of stress-induced increase in β3-adrenergic receptor gene expression in the brain [165]. The stress model used was repeated immune challenge in rats, induced by administration of increasing doses of lipopolysaccharide (LPS). LPS treatment was associated with body weight loss and increased anxiety-like behaviour. In LPS-treated animals of both sexes, β3-receptor gene expression was increased in the prefrontal cortex but not in the hippocampus. Cortical β3-receptors may be related to stress-induced behavioural changes [165].

In conclusion, β3-adrenergic receptors may represent new target also for drugs acting in the brain to induce anxiolytic action. However, potential efforts towards new drug development require additional obtaining and synthesis of knowledge.

## 13. Biomimetic Chromatography as a User-Friendly Technique in Drug Discovery

Tsantili-KakoulidouA.TsopelasF.GiaginisC.

The development of biomimetic chromatography offers valuable support in multi-objective optimization (MOO) approaches, providing a significant advance for rapid experimental–based estimation of ADME properties. Biomimetic properties, defined as the retention outcome of biomimetic chromatography, mimic the biophase either by incorporation (immobilization) of a biological agent in a chromatographic column, or by creation of analogous conditions using appropriate mobile phase additives (surfactants), while they take advantage of the experimental simplicity and reproducibility of chromatographic techniques [166]. They are considered to be positioned in the interface between physicochemical properties (lipophilicity) and in vitro end-points. Biomimetic chromatography incudes immobilized artificial membrane (IAM) chromatography, biopartitioning micellar chromatography, mainly for permeability studies and protein-based chromatography-human serum albumin (HSA) and α1-acid glycoprotein (AGP) chromatography for plasma protein binding studies.

There are two commercially available IAM columns, IAM.PC.MG and IAMPC.DD2, which contain phosphatidylcholine, immobilized on a propylamino-silica gel skeleton. They differ in the end-capping procedure and show comparable performance. Both contain charged centers (phosphate anions and positively charged choline nitrogen) and glycerol carbonyls, which may affect solute retention, by developing electrostatic and hydrogen bonding interactions respectively [166]. However, partitioning in the IAM hydrophobic core remains the main process in retention mechanism. Thus, IAM retention factors encode more complex information than traditional octanol- water distribution coefficients log *D* and may be considered as a border case between passive diffusion and binding to membranes or tissues, a process associated with the concept of phospholipophilicity [166,167]. In most cases IAM retention, expressed by retention factors (log *k*_wIAM_), is not used as a stand-alone parameter and additional descriptors, molecular weight and/or hydrogen bond descriptors are incorporated in the relevant models, always with a negative sign.

We have derived successful non-linear models (R = 0.892) for % human oral absorption (%HOA) for 63 structurally diverse drugs, which are not substrates for uptake or efflux transporters, by combining IAM retention factors (measured at pH 5.5 and 7.4 for bases and acids respectively) with molecular weight, Abraham’s hydrogen bond acidity parameter (both with negative sign) and fraction of protonated species (F^+^). The negative sign of the latter indicates an overexpression of electrostatic interactions of solutes with IAM phosphate anions, which was also evident in IAM retention/liposome partitioning correlation. The model has been successfully validated with a blind test of 22 drugs [168].

In biopartitioning micellar chromatography (BMC) a reversed-phase stationary phase is used. Micelle formation is achieved by adding a surfactant solution above the critical micellar concentration (CMC) as mobile phase additive. Dependent on the type of the surfactant used (neutral, anionic, cationic) different secondary interactions may be developed, while reduced ionization is observed in the micelle environment. Successful application of BMC for human intestinal absorption and for blood-brain barrier permeability has been reported [169].

Protein–based biomimetic chromatography, using a stationary phase with immobilized HSA, offers a friendly alternative for rapid plasma protein binding (PPB) measurements, since HSA is the most important plasma protein [166]. Like for HSA in solution, affinity to HSA column is favoured by acidic drugs. It is well documented that immobilization of HSA does not alter its binding characteristics and many % HSA data, used for screening or QSPR studies, have been determined by HSA-based high-performance liquid chromatography (HPLC). We have demonstrated that isocratic log *k*_10HSA_, measured at 10% acetonitrile as organic modifier in the mobile phase for 52 structurally diverse drugs, produce a 1:1 correlation with affinity constants log *K*, calculated from % PPB [170].

Columns containing the second critical plasma protein, AGP, are also commercially available. They show higher affinity for basic drugs—however, their performance in simulating binding to AGP is not well established. Displacement experiments for cross-linked AGP stationary phases have shown differences in the binding properties as compared to the behaviour of AGP in solution, while the presence of two genetic variants (F1*S and A) is an additional difficulty. We have obtained moderate relationships between AGP retention factors and affinity constants log *K* for F1*S variant or considering the sum of log *K* for both variants, upon introduction of Abraham’s basicity descriptor as additional parameter (R = 0.832 and R = 0.807 respectively [171].

However, we have derived a sound relationship for total plasma protein binding (R = 0.949) combining both HSA and AGP retention factors for 18 structurally diverse, mainly basic, drugs: log *K*(plasma) = 0.77 log *k*_w(HSA)_ + 0.24 log *k*_w(AGP)_ − 0.56. The regression coefficients reflect the different volume ratio of the two proteins in plasma. Beyond passive diffusion, IAM retention factors may also express tissue binding. Ongoing studies indicate that the combination of IAM with HSA retention factors may simulate pharmacokinetic parameters like volume of distribution or unbound fraction in brain, which are related to permeability as well as to protein and tissue binding.

## 14. Chromatography in Analytical Separations of α-Amino Acids

KowalskaT.SajewiczM.

### 14.1. Introduction

**Figure 7:**
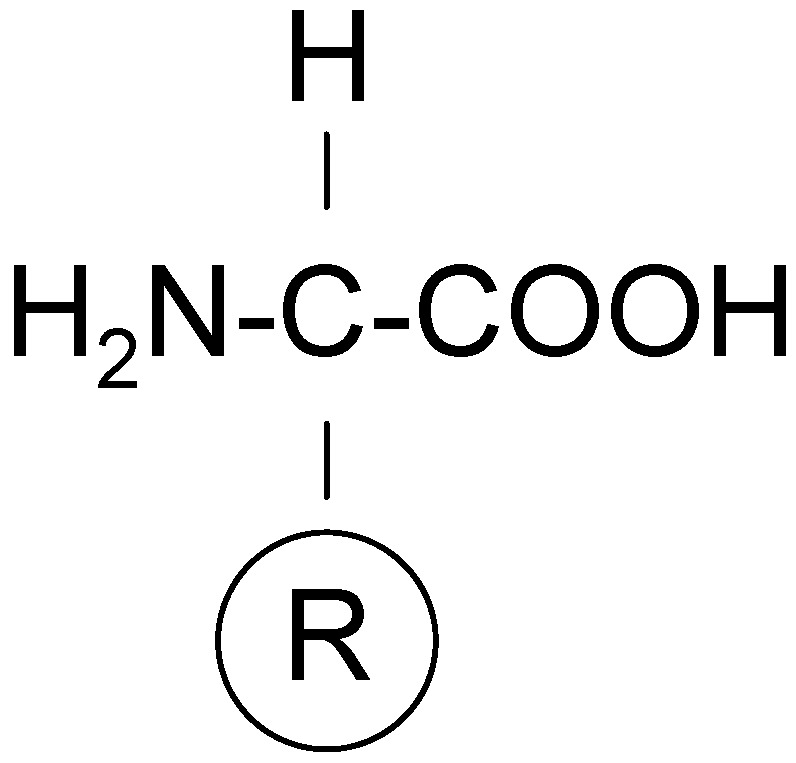
Chemical structure of proteinogenic α-amino acid.

Coded (or primary, or proteinogenic) amino acids include nineteen α-amino acids and one imino acid, and they constitute an alphabet for all proteins. They differ only in the structure of the side chain, R (as indicated in Figure 7) and all of them (except glycine) have the l-configuration. Coded amino acids can be classified according to various different criteria, and one classification system is based on chemical nature of the side chain, R. Thus, we distinguish coded α-amino acids with the non-polar side chain (8 compounds), the neutral polar side chain (7 compounds), and the charged polar side chain (5 compounds). The other group are the non-coded (or non-protein, or non-standard) amino acids that are neither found in proteins assembled during protein biosynthesis, nor generated by posttranslational modifications. Hundreds of such amino acids are known and a large number of these are also α-amino acids. Most non-coded amino acids can be found in plants and microorganisms, and some of them are of great biological importance as peptide antibiotics (e.g., nisin, alamethicin, etc.). There are the four main technological approaches to obtaining the monomeric amino acids on an industrial scale: (*i*) by the extraction of individual proteinogenic α-amino acids from the protein hydrolyzates (for this purpose, the hydrolyzates of human hair as raw material are most frequently used); (*ii*) by chemical synthesis (in these technologies, crude oil is the main substrate); (*iii*) by the procedures with selected enzymes serving as biocatalysts; and (*iv*) by fermentation (in these procedures one needs exclusively sugar and a given bacterium type to obtain a single optically pure α-amino acid) [172,173].

### 14.2. Main Focuses on α-Amino Acids

Proteinogenic α-amino acids have attracted the attention of the scientific world mainly as the simplest building blocks of peptides and proteins, when determination of the primary structure thereof is considered (through the α-amino acid sequencing). As classical chromatographic techniques do not play a key role in decoding the primary structures of peptides and proteins, we will refrain from including this subject matter in our current considerations.

Other important areas of the application of chromatographic techniques to amino acid research are: (*i*) the search for traces of life in extraterrestrial regions; (*ii*) dating of fossilized biological remnants in paleontology; and (*iii*) using amino acids as an inexpensive pool for the enantioresolution studies. In this latter case, either the racemic α-amino acid mixtures represent a resolution problem to be solved, or the optically pure α-amino acids serve as chiral selectors enabling enantioresolution of the other racemic mixtures.

**Figure 8:**
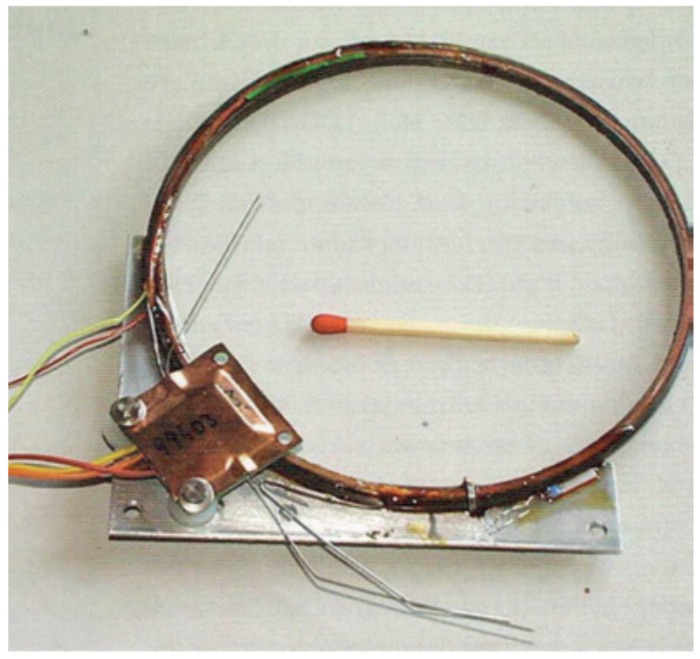
Fused silica capillary column installed on chirality module of the COSAC instrument on board Rosetta’s lander Philae [173].

The search for extraterrestrial traces of life and more precisely, for extraterrestrial samples of proteinogenic α-amino acids has been carried out both on Earth and in the interplanetary regions. Among the most spectacular investigations were those of the huge Murchison meteorite containing over 15 amino acids, some of them representing basic components of life [174]. Another spectacular effort was undertaken by the Rosetta mission of the European Space Agency (ESA), targeting the comet 67P/Churyumov-Gerasimenko (nicknamed Chury). The Philae lander (which after ten years traveling eventually reached the Chury comet on 11 November 2014) was equipped with several miniaturized gas chromatography-mass spectrometry (GC-MS) systems implemented with the gas chromatographic capillary columns prepared to resolve and identify proteinogenic α-amino acids (see Figure 8) [175].

Dating of fossilized biological remnants in paleontology with use of proteinogenic α-amino acids is based on the so-called geochronological clock. Its principle is that with a few important exceptions, living organisms keep all their amino acids in the l-configuration. When an organism dies, control over the configuration of the amino acids ceases, and the ratio of d- to l-moves from a value near 0 towards an equilibrium value near 1, in a process of racemization. The fastest racemizing and hence, the most frequently used α-amino acid is aspartic acid, with its half-life (t_1/2_) estimated as equal to 15.000 ÷ 20.000 years. The concept of geochronological clock was first developed by Bada et al. [176], but later—due to certain inconsistencies in the results obtained with its use—the amino acids racemization data started to be correlated with an outcome of some other dating methods, e.g., based on the kinetics of the ^14^C isotope decay in the deceased and fossilized organisms.

### 14.3. Chromatography in Enantioreolution and Enantioseparation of α-Amino Acids

Among the most challenging tasks in chromatographic analysis is the enantioresolution and enantioseparation of the α-amino acid pairs. Enantioresolution is done in a direct way, using a chiral chromatographic system, whereas enantioseparation is done in an indirect way, using a non-chiral chromatographic system and the pre-column derivatization. In GC, the predominant approach is enantioresolution of a racemate, mainly with use of the three commercially available chiral stationary phase (CSP) types (the hydrogen-bonding type CSPs, the coordination type CSPs, and the cyclodextrin type CSPs). Perhaps it is noteworthy that all three types of the GC stationary phases were used in the Rosetta mission [173]. In HPLC, the predominant approach is enantioseparation with pre-column derivatization, using the Marfey’s reagent (MR) or its analogs [177]. Basic assets of all these derivatization reagents are that their synthesis runs practically stoichiometrically and they practically do not racemize in the course of synthesis. Thin-layer chromatography (TLC) is the most flexible analytical technique out of these three, and it can be used both for enantioresolution and enantioseparation of the racemic α-amino acid mixtures (and of other low molecular weight chiral carboxylic acids), offering a unique potential to devise and in-home prepare various different CSPs of the experimenter’s own choice [177].

### 14.4. Discovery of Spontaneous Oscillatory Reactions

**Figure 9:**
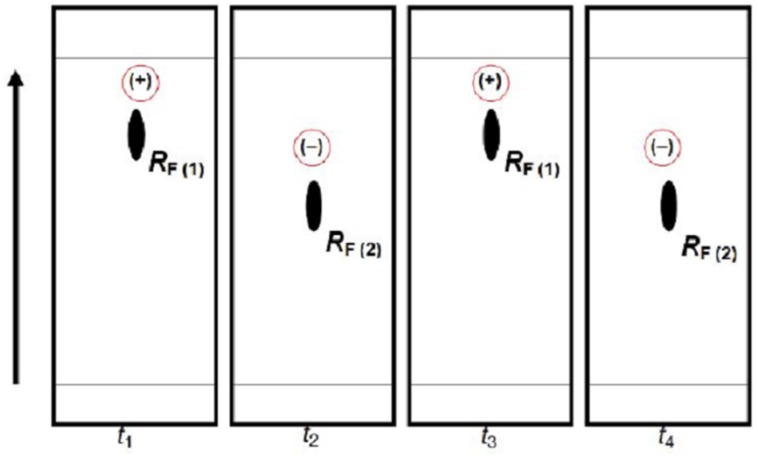
Schematic representation of oscillating changes in *R*_F_ values for low molecular weight chiral carboxylic acids as function of sample storage time (*t*) in solution. With arrow, direction of growing *R*_F_ values is indicated, and *t*_1_, *t*_2_, *t*_3_, and *t*_4_ denote consecutive storage time steps of investigated sample [178].

**Scheme 5:**
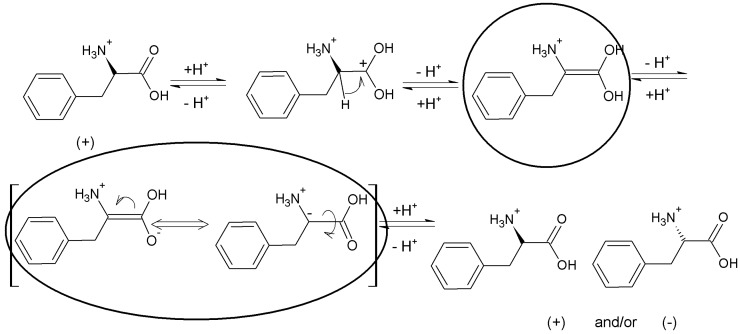
Chiral conversion of l-Phe to d-Phe (intermediary non-chiral structures are marked with black ovals).

An unprecedented performance of TLC as a tool for the enantioresolution of the low molecular weight chiral carboxylic acids (α-amino acids, hydroxy acids and profen drugs) enabled us a discovery of the two novel classes of spontaneous oscillatory reactions, i.e., spontaneous oscillatory chiral conversion and spontaneous oscillatory condensation (in the case of α-amino acids, peptidization (e.g., [178,179,180]). This discovery made with use of TLC was later confirmed by more sophisticated (i.e., highly instrumental) analytical tools. Figure 9 provides schematic representation of the oscillatory changes of a chiral compound’s position on the thin-layer chromatogram in the function of sample storage time (*t*), which correspond with the periodic chiral conversion from (+) to (−) and back. A detailed molecular mechanism of chiral conversion is schematically presented in Scheme 5 upon an example of l-phenylalanine (l-Phe).

### 14.5. Conclusions

Discovery of spontaneous oscillatory chiral conversion and oscillatory peptidization with α-amino acids has a potential to affect the present-day ideas concerning biogenesis and the evolutionary concepts. It certainly undermines the importance of geochronological clock. It clearly shows that a lot more effort has to be invested in future research on physicochemical properties of the monomeric α-amino acids.

## 15. Towards Complete Identification of Drugs of Abuse

SilberringJ.MielczarekP.SmoluchM.

Novel psychoactive substances (NPS) comprise compounds belonging to two major groups, named *Designer drugs* and *Legal highs*. While the first class requires skills in chemical synthesis, the latter can be prepared from the over the counter, legal substances and their preparation can be maintained by an inexperienced teenager, following recipes broadly available on the internet. The scale of seized samples creates an urgent need to develop fast and unambiguous analytical techniques for identification of a large number of such substances in a short time. Moreover, most of such compounds reaching the illegal market [181] generate metabolites of completely unknown function(s) and toxicity on human body. Yet another issue are by-products of various chemical reactions. Adulterants are also frequently added to enhance the strength of a drug or to increase weight of a dose. Altogether, the final effect may lead to serious consequences, including death.

The majority of diagnostic approaches are based on rapid tests, specific for several groups of substances most commonly sold on the market. Examples include cocaine, cannabinoids, opiates, and amphetamines. This indicates that a vast majority of compounds escape selective detection. As it is quite frequent that the drug sold under a given name may contain quite different substance (e.g., Ecstasy/MDMA is estimated to contain other chemicals in ca. 50% deliveries!), therefore there is an urgent need to develop fast, robust, and unambiguous analytical methods to cope with these problems. Additionally, ER admissions for unknown substances constantly increases as they arrive on the market, and patients, as well as ER personnel, are unaware of their content, which cannot be identified by routine analyses.

Here, we propose a general and, in our opinion, complete strategy to solve the above problem. We are also aware, that analytical sciences will always be a step behind skilful chemists synthesizing novel substances. The output, however may at least serve as predictive toxicology approach to more accurately monitor the entire drug profile.

**Figure 10:**
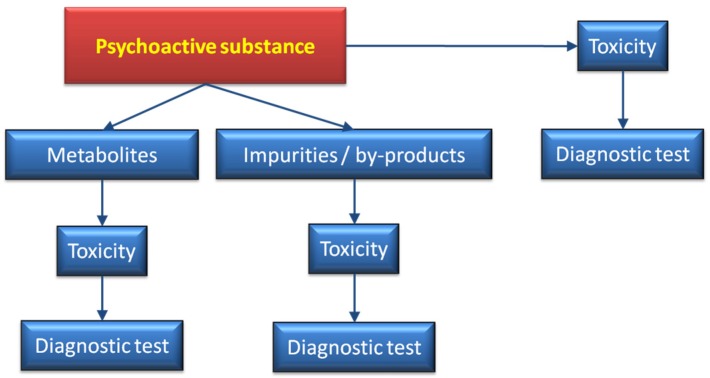
The proposed analytical strategy allowing for complete analytical profile of drug.

Figure 10 shows a scheme where, in addition to the bioactive substance, the metabolites generated in the organism [182], together with adulterants and by-products, are taken into consideration. Bearing in mind that the amount of novel compounds reaching the market (not necessarily illegal) rapidly increases every year. Some of these drugs disappear from the market as soon they are classed as illegal. Thus, it is extremely difficult to cope with the large number of analytical procedures that, in a short time frame may be redundant. A combination of mass spectrometry equipped with ambient ion sources together with fast metabolic studies, may serve as a preliminary approach to assist more detailed work. Among the ion sources that do not require sample preparation are ambient plasma ionization techniques, such as FAPA, DBDI, DART [183]. In vitro generation of potential metabolic products can be achieved within minutes in the electrochemical cell [184], which substantially increases capabilities of the entire analytical procedure. Such products generated by electrochemistry may be screened for eventual toxicity using various cell cultures. The entire analytical strategy should be perceived as an initial approach, prior to more advanced research.

## 16. Application of XRD and DSC Methods for Identification of Counterfeit Drugs

JendrzejewskaI.

A significant increase in the availability of drugs, especially the possibility to purchase medical preparations in shops, at petrol stations or on the Internet, creates the possibility for introducing counterfeit drugs, which can contain inappropriate substances, abnormal amounts of active substances or significant amount of impurities. Counterfeit pharmaceuticals may threaten health and life, and for this reason, it is important to monitor and investigate compositions of pharmaceutical materials using various techniques, such as diffraction, thermal analysis, spectroscopy, etc. The combination of two or more investigative methods allows for confirmation of the authenticity of a drug.

**Figure 11:**
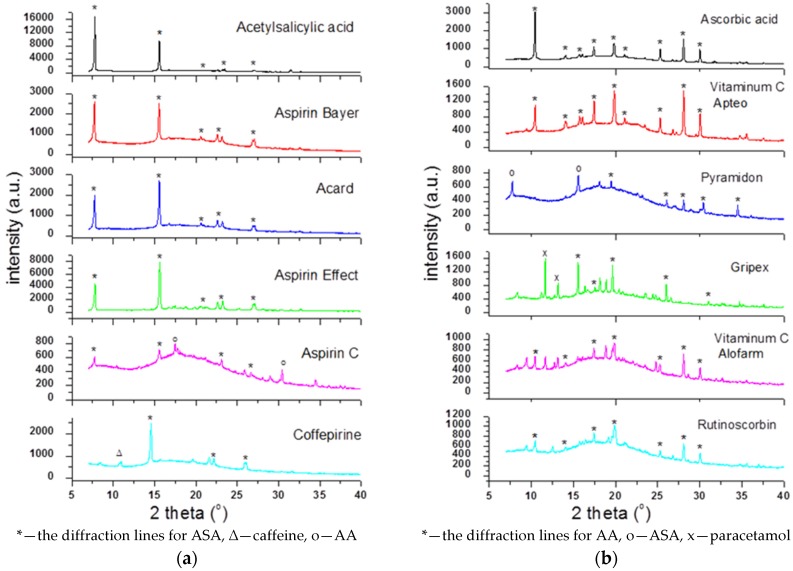
Diffraction patterns for drugs containing (**a**) acetylsalicylic acid (ASA); (**b**) ascorbic acid (AA).

To check the presence of API (active pharmaceutical ingredient) in common drugs and dietary supplements, a combination of PXRD (powder X-ray diffraction) and DSC (differential scanning calorimetry) methods was used. For this study, the selected over-the-counter medicines and dietary supplements containing acetylsalicylic acid and ascorbic acid were chosen. The PXRD is a fast, reliable and easy to use technique, which can be used in forensic science to analyse various types of trace evidence [185]. Most often, the X-ray powder diffraction is used for phase identification of crystalline materials and to gain information about structural parameters [186]. Crystalline materials have distinct PXRD spectra and can be identified within a compound or mixture using a database of known PXRD spectra [187]. Thermal analysis is a branch of materials science where the properties of materials are studied as they change with temperature. The differential scanning calorimetry measures the rate of heat flow to a sample and a standard that are at the same temperature. The PXRD measurements were carried out using a Philips PW1050 diffractometer, whereas the DSC measurements were done using a DSC-TG Labsys Evo system. These samples were investigated in a polycrystalline form. For the interpretation of the obtained diffraction patterns, the X-ray structural analysis based on ICDD PDF-2 was used [188]. The PXRD analysis confirmed that in all investigated samples, the active APIs (acetylsalicylic acid (ASA) and ascorbic acid (AA)) was present. For some medicines, the diffraction lines of other APIs were observed in their diffraction patterns (Figure 11).

**Figure 12:**
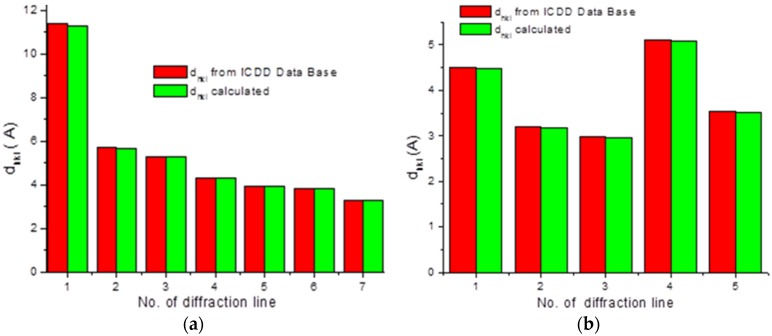
Comparison of calculated values of d_hkl_ with values found in the ICDD Data Base: (**a**) Aspirin^®^ (Bayer); (**b**) vitamin C (Apteo).

The phase analysis was done based on ICDD Cards: No. 00-001-0182 for ASA and No. 00-022-1559 for AA. The diffraction patterns are best reported using interplanar distance d_hkl_ rather than the value of 2θ angle. The peak position at 2θ angle depends on instrumental characteristic such a wavelength. The peak position as d_hkl_ is an intrinsic, instrument-independent, material property. The Bragg’s Law was used to convert observed 2θ position to d_hkl_:d_hkl_ = nλ/2sinθ (where n—diffraction order, λ—X-ray wavelength, θ—angle of reflection). The values of d_hkl_ for the studied samples are in good accordance with those presented in the ICDD database (Figure 12).

**Figure 13:**
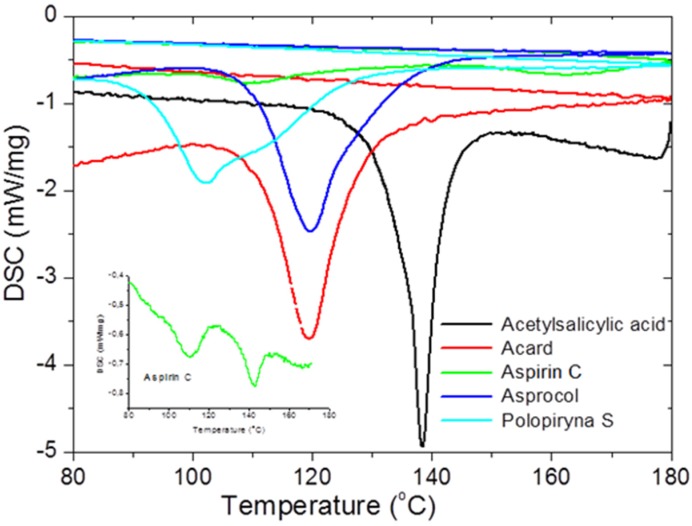
DSC curves for drugs containing acetylsalicylic acid.

The intensities of the diffraction lines depend on the content of the component in the tested preparation. Therefore, different intensities of lines for the APIs were observed in the obtained diffraction patterns. The thermal analysis was carried out for drugs containing ASA. This analysis showed a weight loss of about 5%, which can indicate a thermal dissociation with a loss of CO_2_ or H_2_O, or emission of gaseous products [189]. The observed shift for the endothermic peak in the DSC curves, towards a lower temperature, indicates the presence of other components (i.e., excipients) or impurities in the investigated samples in comparison with pure acetylsalicylic acid (Figure 13).

Based on the above investigation, it can be stated that a combination of the two methods: XRD and DSC can be used to distinguish original drugs from counterfeit products, e.g., by checking for the presence of the correct API or by a comparison of the drugs fingerprint.

## 17. Big Data Problem in Drug Design

PolanskiJ.

It is generally believed that big data brings new value and innovation. For example, Szlezak et al. cited the recent McKinsey research that suggests that the potential use of the big data in the US health care could reduce costs by $300 billion a year [190]. Accordingly, do we need big data in drug design and does this method bring new prospects into pharmaceutical business? The answer seems to be positive. However, despite high expectations the number of big data approaches in drug design are surprisingly low.

What is big data and how important is big data in drug design? What are the methods that are used for big data analyses? The structure of big data that are used in drug design has been recently analysed by Polanski [191] who indicated several basic big molecular data types. Basically, these are the big data that are generated by descriptor (DE) or property (PE) expansion. There are several typical architectures of potential big data analyses in drug design:

1. The expansion of the number of descriptors typical in QSAR investigations;
2. The analyses of all available property data. Typical examples are IC_50_ vs. MW statistics for all pharmaceutics or drug candidates;
3. The expansion of the number of properties; e.g., in the polypharmacology concept.

QSAR (ad point 1) operates on a well described data architecture usually generating a number of descriptors for a compound series of 20–100 compounds with a known activity. The analysis of the complete population, i.e., *no sampling*, meets one of the requirements of big data. Molecular populations in drug design are limited, e.g., we have 1564 approved drugs, 1836 targets, 11,387 drug-target interactions (DrugBank 5.0.3) [192]. Accordingly, although the analysis of *all possible data* in the architecture (ad point 2) does not focus on extremely high numbers, to some extent this reminds big data. Molecular statistics is a term that can describe such analyses [193]. Molecular statistics can result in important conclusions in drug design, such as for example:

- Commercial drugs usually have lower MW that drug candidates; this is known under the molecular obesity or slim pharma concept [194,195];
- There is no correlation between IC_50_ of drugs and their therapeutic dose [196];
- The majority of commercial drugs in new classes are developed by phenotypic (new classes) or target based (known classes) screening [197];
- The IC_50_ of drugs is similar to normal distribution [198];
- IC_50_ of drug candidates (CHEMBL) increases with the increase of MW and log *P* [196];
- Ligand efficiency [195] does not provide a measure of the ligand-target binding power but is dominated by the Avogadro type statistics determining a number of molecules in a 1 g sample [199,200].

Eventually, real big data in drug design needs measured property data. Properties, however, are seldom available. This means that the PE data are usually getting bigger not by the number of property types but by an increase in the number of chemical compounds that are annotated with a single property type. However, even such data are usually limited in numbers, e.g., in a virtual HTS screening of 1.5 million real commercial compounds recorded in Enamine database a set of 1140 compounds with determined HIV-1 IN inhibition fetched from the ChEMBL v.12 has been used for guiding the activity pattern [65]. We should realize from the definition of big data by Oxford Dictionaries that data are getting especially bigger while recording human behaviour and interactions. This can be understood when we compare a population of the factually registered molecules, i.e., ca. 150,000,000 chemical compounds, with the much larger population of humans counting ca. 7 billion people. This decides that the human population provides more complex data then the molecular one. Accordingly, although the relationship between a chemical structure and its physical or chemical properties is an essential concept in chemistry, it is the market formed by human interactions that brings big data and eventually decides the success of any pharmaceutical. Therefore; we need economic considerations to fully understand a fate of a drug. Economic behaviour, in particular, a price of a drug is an example of the nonbinding parameter important for molecular design. Is there any relationship between a chemical structure of a drug and its economic potential? On the one hand, explaining economic effects is an extremely complex issue. On the other hand, little market data is available for drugs. The problem remains unexplored. Instead, a structure-economy relationship method has been recently developed to analyse a big data for a large library of 2.5 million synthetic building blocks [193].

Formally, all the examples discussed above are architectures of the type (ad point 2). It is however, architecture (ad point 3) that brings a real breakthrough in the design opportunity. In this context, the polypharmacology inspired scheme of lipidomics has been developed recently by Gabriele Cruciani group [201]. The method is limited by an inexpensive and rapid procedure for the extraction and MS based determination of a medium size population of lipid species that can characterize the complex answer of any organism for a drug (drug candidate) administration. In 20 min this provides us a data on the serum concentration of 1000 different lipids in serum. Such a data can be arranged in a fingerprint-like lipid profile which if tested as a function of time, can give us a real big data presenting useful information on the activity and toxicity of the xenobiotics used. This illustrated that more efficient big data investigations are limited not necessarily by the statistical issues but by new methods in inexpensive property measurements [202]. New HTS methods for property measurements should undoubtedly bring new quality in big data analyses in drug design.

## Figures and Tables

**Table 1:** Selected activities of styrylquinolines **B** (R=H, see Scheme 4) against wild type and p53 *null* colorectal cancer cells [73].

Compounds		Activity IC_50_ [μM]		
No.	R^1^	HCT116 (p53^+/+^)	HCT116 (p53^−/−^)	Therapeutic index *
CP-31398		18.63 ± 0.92	26.28 ± 1.41	0.4
B	2,3-Cl	5.13 ± 1.41	2.99 ± 0.61	>20
B	3,4-Cl	9.41 ± 2.17	3.34 ± 0.58	>8
B	2-F	15.43 ± 2.46	8.38 ± 1.12	>3

* calculated as IC_50_p53^−/−^ to normal fibroblasts (GM07492).
